# Inhibition of DNA methyltransferase 1 increases nuclear receptor subfamily 4 group A member 1 expression and decreases blood glucose in type 2 diabetes

**DOI:** 10.18632/oncotarget.10043

**Published:** 2016-06-14

**Authors:** Yng-Tay Chen, Jiunn-Wang Liao, Ya-Ching Tsai, Fuu-Jen Tsai

**Affiliations:** ^1^ Human Genetic Center, Department of Medical Research, China Medical University Hospital, China Medical University, Taichung, Taiwan; ^2^ Graduate Institute of Veterinary Pathobiology, Research Center for Animal Medicine, Animal Disease Diagnostic Center, National Chung Hsing University, Taichung, Taiwan; ^3^ Graduate Institute of China Medical Science, China Medical University, Taichung, Taiwan; ^4^ Department of Medical Genetics, China Medical University Hospital, Taichung, Taiwan; ^5^ School of Chinese Medicine, China Medical University, Taichung, Taiwan; ^6^ Department of Health and Nutrition Biotechnology, Asia University, Taichung, Taiwan

**Keywords:** type 2 diabetes, DNA methylation, NR4A1, DNMT1, epigenetics, Gerotarget

## Abstract

Our previous genome-wide association studies showed that DNA methyltransferase 1 (DNMT1) is associated with increased susceptibility to type 2 diabetes (T2D) in Han Chinese individuals. Here, we aimed to further evaluate the role of DNMT1 in T2D. We performed a genome-wide DNA methylation array and found that the nuclear receptor subfamily 4 group A member 1 (*NR4A1*) promoter was hypermethylated in patients with T2D and in a mouse model of T2D. Moreover, DNA hypermethylation of the *NR4A1* promoter reduced *NR4A1* mRNA expression. Transient transfection of human NR4A1 into RIN-m5F and 293T cells caused DNMT1 inhibition and induced insulin receptor activation. NR4A1knockdown by shRNA resulted in overexpression of DNMT1 and inhibition of insulin receptor, suggesting that the *NR4A1* gene is involved in the epigenetics pathway. Furthermore, T2D model mice treated with the DNMT1 inhibitor aurintricarboxylic acid (ATA) showed reduced activation of DNMT1 in pancreatic β cells; this effect reversed the changes in NR4A1 expression and decreased blood glucose in T2D model mice. Thus, our results showed for the first time that DNMT1 caused *NR4A1* DNA hypermethylation and blocked insulin signaling in patients with T2D. Importantly, ATA therapy may be useful for decreasing blood glucose levels by reversing NR4A1-dependent insulin signaling. These findings improve our understanding of the crucial roles of these regulatory elements in human T2D.

## INTRODUCTION

Type 2 diabetes (T2D) is a complex disease that is characterized by hyperglycemia, pancreatic β-cell dysfunction, decreased insulin signaling, and increased hepatic glucose production [1–4]. We previously identified the rs78789647 T allele in the DNA methyltransferase 1 (*DNMT1*) gene as the allele having the strongest association with T2D in Han Chinese individuals by epigenetic regulation of the protein tyrosine phosphatase receptor type delta (*PTPRD*) gene in the insulin signaling pathway [5–7]. Additionally, in the pancreas, DNA methylation is involved in regulating de novo β-cell formation [8, 9]. Recent studies have shown that these epigenetic factors contribute to the pathogenesis of T2D. However, the specific mechanisms through which DNMT1 regulates T2D are unclear, and the roles of DNMT1 in T2D onset and development have not been elucidated.

Nuclear receptor subfamily 4 group A (NR4A) family receptors are expressed as early response genes of physiological and pathological stimuli, including fatty acids, stress, prostaglandins, growth factors, calcium and inflammatory cytokines, peptide hormones, and neurotransmitters [10], and are implicated in important biological processes, including carcinogenesis, apoptosis control, inflammation, vascular disease, dopaminergic neuron development, and metabolism [11]. The NR4A member 1 (*NR4A1*) gene, which has been shown to be associated with insulin in cell models [9], encodes is a transcriptional regulator of glucose metabolism in liver and skeletal muscle [12–15].

Genome-wide DNA methylation arrays have become important in evaluation of epigenetic changes associated with disease progression. Here, we performed a pilot study using a DNA methylation array with samples from patients with T2D in order to identify biomarkers associated with the onset and development of T2D in Taiwan. Moreover, to gain insights into the functions and mechanisms through which DNMT1 affects the insulin signaling pathway, we examined epigenetic changes in patients with T2D and in a mouse model of T2D.

## RESULTS

### *NR4A1* promoter hypermethylation in patients with T2D

**Figure 1:**
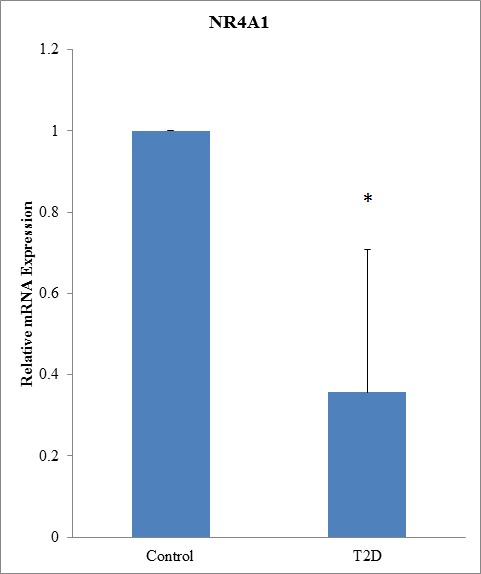
*NR4A1* mRNA was downregulated in patients with T2D mRNA from 94 patients with T2D and 98 normal controls was used for qRT-PCR. Expression levels are shown relative to that in control patients (*P* < 0.05).

First, we performed a genome-wide DNA methylation array of samples from patients with T2D. DNA methylation array data are accessible via Gene Expression Omnibus database (GEO), accession number GSE81868 (http://www.ncbi.nlm.nih.gov/geo/query/acc.cgi?acc=GSE81868). Analysis of DNA methylation status, as indicated by model-based analysis of tiling-arrays (MAT) scores (range: 5.03684-8.45898), showed that the following genes had the highest methylation scores: *ABR*, *ERICHI*, *WDFY2*, *GUSBP1*, *NR4A1*, *AFF2*, *PIPK1B*, *LOC100288637*, *APBA1*, and *PTPRN2* (Table 1). Further analysis of blood samples from patients with T2D and healthy controls showed that relative *NR4A1* mRNA levels were lower in patients with T2D than in controls (1 versus 0.356, respectively; *P* < 0.05; Figure 1).

**Table 1:** Top 10 significant differentially hypermethylated genes in human T2D *versis* CTL

Transcript ID	Gene Symbol	MAT-score^a^	*p*-value
NM_002847	PTPRN2	8.45898	8.79E-06
NM_001163	APBA1	5.5031	8.79E-06
NR_038253	LOC100288637	5.40479	8.79E-06
NM_001278253	PIP5K1B	5.39986	8.79E-06
NM_001169122	AFF2	5.22154	8.79E-06
NM_001202233	NR4A1	5.14054	8.79E-06
NR_027026	GUSBP1	5.11848	8.79E-06
NM_052950	WDFY2	5.08292	8.79E-06
NR_073397	ERICH1-AS1	5.0794	8.79E-06
NM_001159746.1	ABR	5.03684	8.79E-06

a MAT-score: Model-based Analysis of Tiling-arrays-score

### NR4A1 was involved in the insulin signaling pathway

**Figure 2:**
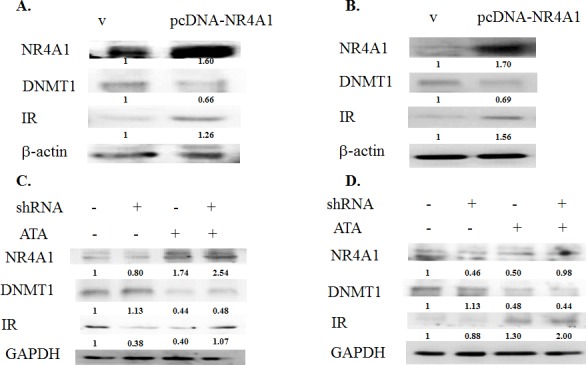
The *NR4A1* gene was epigenetically regulated in the insulin signaling pathway **A.** 293T cells were transfected with the pcDNA vector or pcDNA-NR4A1 for 48 h, and the effects of NR4A1 overexpression on DNMT1 inhibition and induction of insulin receptor (IR) overexpression were analyzed. **B.** RIN-m5F cells were transfected with NR4A1 for 48 h, and the effects of NR4A1 overexpression on DNMT1 inhibition and induction of IR expression were analyzed. **C.** Effects of NR4A1 knockdown by shRNA and treatment with ATA in 293T cells. **D.** Effects of NR4A1 knockdown by shRNA and treatment with ATA in RIN-m5F cells.

The role of NR4A1 in the insulin signaling pathway is still unclear. Therefore, we used an *in vitro* model to analyze the effects of NR4A1 expression on insulin signaling. A plasmid containing a fragment of human NR4A1 (pcDNA-NR4A1) was constructed and transiently transfected into 293T and RIN-m5F cells. The results indicated that expression of human NR4A1 inhibited the activity of DNMT1, but induced insulin receptor overexpression in cells (Figure 2A, 2B), and DNMT1 and NR4A1 affected glucose-stimulated insulin secretion (GSIS; Figure S1), suggesting that NR4A1 was involved in the insulin signaling pathway and affected by DNMT1.

### Effects of DNMT1 inhibition on NR4A1 expression

Interestingly, knockdown of NR4A1 expression by shRNA in 293T and RIN-m5F cells resulted in simultaneous downregulation of the insulin receptor and induction of DNMT1 in RIN-m5F cells. These results further supported that NR4A1 was involved in the insulin signaling pathway and affected by DNMT1. Therefore, we treated cells with the DNMT1 inhibitor aurintricarboxylic acid (ATA). The results showed that ATA induced NR4A1 expression in not only RIN-m5F cells and 293T cells but also NR4A1-knockdown cells. In addition, the insulin receptor was induced in RIN-m5F and 293T cells (Figure 2C, 2D).

### ATA decreased blood glucose and induced changes in b-cells

**Figure 3:**
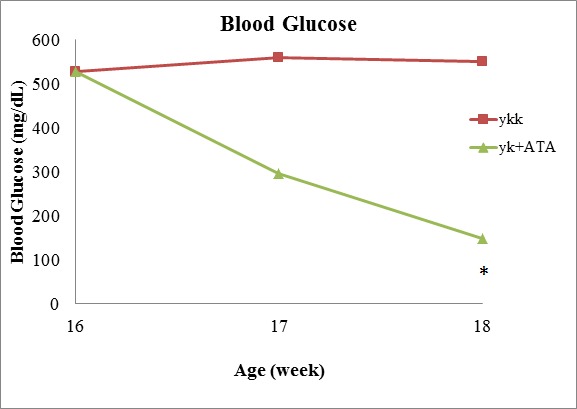
ATA decreased blood glucose levels in T2D model mice Sixteen-week-old mice were treated with ATA daily, and blood glucose was measured after 2 weeks.

**Figure 4:**
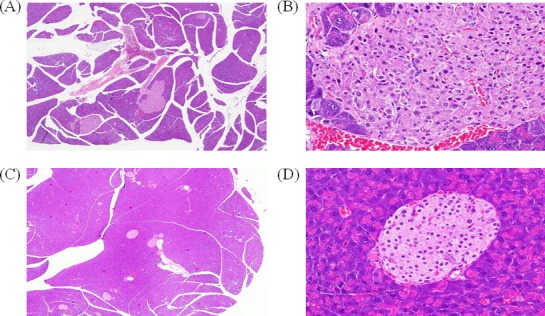
Effects of ATA on pancreatic islet mass H&E staining of pancreas islet mass after 2 weeks of ATA treatment. **A.** Pancreas samples from ^y^KK mice, 100×. **B.** Pancreas samples from ^y^KK mice, 400×. **C.** Pancreas samples from ATA-treated ^y^KK mice, 100×. **D.** Pancreas samples from ATA-treated ^y^KK mice, 400×.

**Figure 5:**
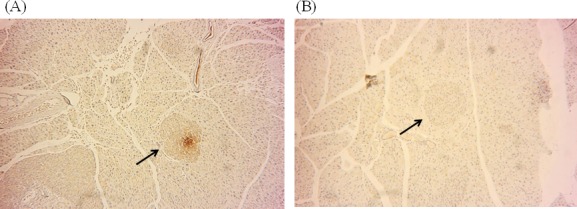
DNMT1 expression was inhibited by ATA in the pancreas **A.** Immunocytochemical staining of pancreas tissues showing DNMT1 protein expression in ^y^KK mice. **B.** Expression of DNMT1 in the pancreas after ATA treatment for 2 weeks.

**Figure 6:**
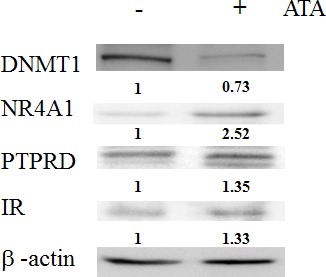
Effects of ATA in the insulin signaling pathway Expression levels of DNMT1, NR4A1, PTPRD, and insulin receptor (IR) in livers from T2D model mice.

Next, we used a mouse model of T2D to further elucidate the role of DNMT1 in diabetes. Sixteen-week-old mice showing insulin resistance were treated with ATA daily for 2 weeks. The results showed that blood glucose was significantly lower in ATA-treated T2D mice than in control mice (149.3 versus 526.7 mg/dL, respectively; *P* < 0.05; Figure 3). In ^y^KK mice, which were used in this study, insulin resistance is associated with hypertrophy of pancreatic islets and degranulation of β-cells. After ATA treatment, pancreas islets showed decreased mass (Figure 4) and DNMT1 inhibition (Figure 5). Moreover, NR4A1 DNA hypermethylation was reduced by ATA (Figure S2), insulin signaling was triggered by insulin receptor activation, PTPRD induction, and NR4A1 overexpression (Figure 6).

## DISCUSSION

Genome-wide DNA methylation profiling data can reveal markers that may explain clinical and pathological specifics of T2D in a given population. DNA methylation may also have applications as a diagnostic and therapeutic target in patients with T2D [16]. In this study, we found that *NR4A1* DNA was hypermethylated, resulting in downregulation of *NR4A1* mRNA in patients with T2D. A recent microarray analysis indicated that NR4A1 does not induce genes associated with cell stress or apoptosis, suggesting that this protein may function mainly to stimulate proliferation when activated in the context of islet β-cells [17, 18]. Genetic deletion of NR4A1 increases susceptibility to diet-induced obesity and insulin resistance, and loss of NR4A1 expression in skeletal muscle impairs insulin signaling, markedly reduces GLUT4 protein expression, and increases triglyceride content [19]. Thus, our report presents the first findings to support the mechanism of NR4A1 function in T2D.

NR4A1 has been shown to be induced by insulin and thiazolidinedione drugs in 3T3-L1 adipocytes, and *NR4A1* gene expression is enhanced in skeletal muscles and adipose tissues in multiple rodent models of insulin resistance [9]. Consistent with these findings, our results showed that the *NR4A1* gene was epigenetically regulated by DNMT1 and was involved in the insulin signaling pathway, providing support for the participation of NR4A1 in pancreatic insulin receptor signaling.

In a previous study, we found that the *DNMT1* gene was associated with susceptibility to T2D in Han Chinese individuals. Importantly, *DNMT1* expression is significantly upregulated in mice and humans with T2D compared with that in controls [7]. Moreover, a single nucleotide polymorphism in *DNMT1* (rs78789647) has been shown to be correlated with susceptibility to T2D, and DNMT1 has a preference for hemimethylated DNA [20–22].

KK-A^y^ mice carry the lethal yellow obese (A^y^) mutation and develop diabetes of polygenic origin, showing severe obesity, hypertriglyceridemia, hyperglycemia, hyperinsulinemia, and insulin resistance by 16 weeks of age [23, 24]. In the current study, we treated 16-week-old ^y^KK mice with ATA, which has been shown to inhibit DNMT1 [25]. Importantly, ATA treatment caused decreased blood glucose, increased NR4A1 expression, PTPRD recovery, and insulin receptor activation, providing further insights into the effects of ATA on T2D and DNMT1 function.

Hepatic expression of NR4A1 is induced by the cAMP axis in response to glucagon and fasting *in vivo* and is increased in diabetic mice exhibiting elevated gluconeogenesis [14]. Our previous report showed that PTPRD was inhibited by DNMT1 overexpression in patients with T2D and T2D model mice [7]. Moreover, inhibition of DNMT1 activity with 5-aza-2′-deocycytidine may facilitate the reprogramming of mouse embryonic fibroblasts into pluripotent stem cells [26], and drugs that seem to be inefficient in solid tumors could be applicable to particular subgroups of patients with pancreatic ductal adenocarcinoma [27]. Another group suggested that genomic hypomethylation caused by disrupted DNMT1 activity is correlated with a greater capability to form de novo beta cells in response to ablation [8]. Thus, our results showed, for the first time, that NR4A1 was induced by DNMT1 inhibition, resulting in insulin receptor activation and decreased blood glucose.

In summary, we found that NR4A1 expression was significantly lower in patients with T2D. Additionally, NR4A1 was involved in the insulin signaling pathway and could be epigenetically silenced by DNA methylation; epigenetic recovery of NR4A1 was observed following DNMT1 inhibition. We also found that the DNMT1 inhibitor ATA functioned to regulate blood glucose in T2D. These findings improve our understanding of the crucial roles of various regulatory elements in human T2D susceptibility.

## PATIENTS AND METHODS

### Isolation of methylated DNA by MBDCap

A MethylMiner Methylated DNA Enrichment Kit (Invitrogen, Carlsbad, CA, USA) was used to isolate methylated DNA. Genomic DNA was extracted from peripheral blood leukocytes using a Genomic DNA extraction kit (Qiagen, Valencia, CA, USA), according to the manufacturer's instructions. One microgram of genomic DNA was sonicated to produce fragments ranging from 100 to 500 bp. Then, 3.5 mg (7 mL) of MBD-Biotin Protein was added to 10 mL Dynabeads M-280 Streptavidin according to the manufacturer's instructions. The MBD-magnetic bead conjugates were washed three times and suspended in 1 volume of 13 bind/wash buffer. The capture reaction was performed by adding 1 mg sonicated DNA to the MBD-magnetic beads on a rotating mixer for 1 h at room temperature. All capture reactions were carried out in duplicate. The beads were washed three times with 13 bind/wash buffer. The methylated DNA was eluted in one of the following two ways: (1) as a single fraction with a high-salt elution buffer (2000 mM NaCl), denoted as MBD-SF; or (2) as distinct subpopulations based on the degree of methylation using an increasing NaCl concentration of the elution buffer, from 200 to 2000 mM in a stepwise gradient (elution 1, 200 mM; elution 2, 350 mM; elution 3, 450 mM; elution 4, 600 mM; elution 5, 1000 mM; and elution 6, 2000 mM). Each fraction was concentrated by ethanol precipitation using 1 mL glycogen (20 mg/mL), 1/10th volume of 3 M sodium acetate (pH 5.2), and two sample volumes of 100% ethanol and was subsequently resuspended in 60 mL H_2_0.

### Promoter array analyses

Immunoprecipitated DNA and input DNA from MeDIP immunoprecipitations and MBD-Capture reactions were amplified with a GenomePlex Single Cell Whole Genome Amplification Kit (cat. no. WGA4; Sigma, St. Louis, MO, USA), according to the manufacturer's instructions. Fifty nanograms of DNA was used in each amplification reaction. The reactions were cleaned up using cDNA cleanup columns (cat. no. 900371; Affymetrix), and 7.5 μg of amplified DNA was fragmented and labeled according to Affymetrix Chromatin Immunoprecipitation Assay Protocol P/N 702238 Rev. 3. Affymetrix GeneChip Human Promoter 1.0R arrays (P/N 900777) were hybridized using a GeneChip Hybridization wash and stain kit (P/N 900720).

Hybridization was performed with an Affymetrix GeneChip Promoter 1.0R array. The arrays were hybridized for 17 h at 45°C and 60 rpm. Arrays were subsequently washed (Affymetrix Fluidics Station 450), stained with streptavidin-phycoerythrin (GeneChip Hybridization, Wash, and Stain Kit; cat. no. 900720), and scanned on an Affymetrix GeneChip Scanner 3000.

### Patients and sample collection for quantitative real-time polymerase chain reaction (qRT-PCR) analysis

A total of 192 blood samples (98 from healthy controls and 94 from patients with T2D) were collected. All individuals attended the China Medical University Hospital in Taichung, and patients fulfilled the diagnostic criteria for T2D. Total RNA was isolated from human blood using a High Pure RNA Isolation Kit (Roche, Mannheim, Germany) according to the manufacturer's instructions. cDNA was synthesized from 1 μg total RNA using a High Capacity cDNA reverse transcription kit (Applied Biosystems, Foster City, CA, USA), in a 20-μL reaction volume, according to the manufacturer's instructions. cDNA was diluted to 10 ng/L, and 1 μL cDNA was used for each qRT-PCR assay in a final reaction volume of 10 μL. For quantification of gene expression with the ABI ViiA 7 Real-Time PCR System (Applied Biosystems), FastStart Universal SYBR Green Master mix (Roche) was used. Primer sequences were as follows: *NR4A1* sense: 5′-TCTATGTCCTCGCCTTGGTT-3′, antisense: 5′-ATTATCCCGTCTGCCTTCAG-3′; *GAPDH* sense: 5′-CAGCCTCAAGATC ATCAGCA-3′, antisense: 5′-TGTGGTCATGAGTCCTTCCA-3′. This study was approved by the Human Studies Committee of China Medical University Hospital, and informed consent was obtained from either the participants or their parents.

### Transfection

RIN-m5F rat pancreatic β-cells and 293T cells were purchased from Food Industry Research and Development Institute (Hsinchu, Taiwan). Cells were seeded at 150,000 cells per well in six-well culture plates and incubated until the culture reached 50-80% confluence. Cells were then transfected with either the empty pcDNA3.0 vector, pcDNA3.0-NR4A1 construct, or lentiviral expression system for NR4A1 shRNA (provided by the National RNAi Core Facility, Academia Sinica, Taiwan) using Xfect Transfection Reagent (Clontech, Palo Alto, CA, USA), according to the manufacturer's instructions. At 48 h after transfection, total protein was isolated from the cells.

### Protein extraction and western blotting

The cells were homogenized in ice-cold RIPA lysis buffer (Millipore, Temecula, CA, USA) with freshly added protease inhibitor and phosphatase inhibitor (FIVEphoton, San Diego, CA, USA). The homogenate was incubated on ice for 30 min and centrifuged at 13,000 × *g* for 30 min at 4°C. The supernatant was used for western blotting. Proteins in a 40-μg sample of the supernatant fraction were separated by sodium dodecyl sulfate polyacrylamide gel electrophoresis (SDS-PAGE) using a 10% acrylamide resolving gels and transferred to polyvinylidene difluoride membranes. Membranes were incubated in blocking solution containing 5% nonfat dry milk and 0.1% Tween-20 in Tris-buffered saline, followed by incubation with rabbit anti-NR4A1 (1:1,000; LifeSpan BioSciences, Seattle, WA, USA), anti-DNMT1 (1:1,000; LifeSpan BioSciences), anti-insulin receptor (1:1,000; GeneTex, Irvine, CA, USA), anti-PTPRD (1:1,000; GeneTex), and anti-actin (1:5,000; GeneTex) polyclonal primary antibodies. Membranes were then incubated with horseradish peroxidase-conjugated goat anti-rabbit IgG (1:5,000; Jackson ImmunoResearch, West Grove, PA, USA) secondary antibodies. Proteins were visualized using SuperSignal West Pico Chemiluminescent Substrate or SuperSignal West Femto Chemiluminescent Substrate (Thermo, Rockford, IL, USA).

### Animals

Four-week-old male KK and KK.Cg-*A^y^*/J mice were obtained from Jackson Laboratories (Bar Harbor, ME, USA). Sixteen-week-old mice were treated intraperitoneally with ATA (50 mg/kg/day) for 2 weeks. Animals were housed in individual cages and given lab chow *ad libitum* (LabDiet 5k52; St. Louis, MO, USA). The animals were housed in a room with a constant temperature (22-25°C), relative humidity (50-70%), and photoperiod (12-h light/12-h dark). This study was approved by the Institutional Animal Care and Use Committee (IACUC) of China Medical University (IACUC: 102-217).

### Immunohistochemistry

DNMT1 was performed using rabbit anti-DNMT1 antibodies (LifeSpan BioSciences) and a streptavidin-biotin-peroxidase complex for immunohistochemistry staining. Formalin-fixed paraffin-embedded tissue sections were incubated in 3% H_2_O_2_ in distilled water for 30 min at room temperature, followed by antigen retrieval by boiling the sections in 0.01 M citrate buffer for 20 min for deparaffinization and rehydration. Sections were then washed in 50 mM Tris-HCl (pH 7.6) with 0.05% Tween for 2 min. To block nonspecific binding, all sections were incubated with 5% nonfat dry milk for 30 min at room temperature. The slides were then incubated with anti-DNMT1 antibodies (1:250) for 1 h at room temperature. The reaction was stopped by rinsing the sections with 0.01 M PBS. The sections were then incubated with biotinylated anti-mouse/rabbit IgG serum (secondary antibody), followed by treatment with a peroxidase-labeled streptavidin-biotin complex and diaminobenzidine substrate to visualize the positive cells. Finally, sections were counterstained with hematoxylin, prior to being mounted for examination by light microscopy. DNMT1-positive cells per area in the mouse pancreas were counted using a Q500MC Image Analysis System (Leica, Nussloch, Germany). DNMT1-positive cells were quantified in 20 fields from the cortex and 15 fields from the medulla at a magnification of 200×.

### Statistical analysis

Data were organized using Microsoft Excel software and analyzed using SPSS 15.0 (SPSS, Chicago, IL, USA) or GraphPad Prism version 3 (GraphPad Software, San Diego, CA, USA). All values were expressed as means ± standard deviations. Normality of the data was tested using the Kolmogorov-Smirnov test. Hierarchical gene analysis and heat maps were determined using a Pearson correlation matrix. Depending on the probability distribution pattern and the total number of subjects, parametric (normal distribution and ≥ 50 subjects) or nonparametric tests (skewed distribution or < 50 subjects) were used. The level of significance was set at *P* < 0.05 (two-tailed).

## SUPPLEMENTARY MATERIALS FIGURES


