# Supplementary material for: Inhibition of DNA methyltransferase 1 increases nuclear receptor subfamily 4 group A member 1 expression and decreases blood glucose in type 2 diabetes
Source: Oncotarget. 2016 Jun 14;7(26):39162–70. doi: 10.18632/oncotarget.10043 (PMC5129922; doi:10.18632/oncotarget.10043)
Supplement: Supplementary file 1 [file oncotarget-07-39162-s001.pdf]

Inhibition of DNA methyltransferase 1 increases nuclear receptor subfamily 4 group A member 1 expression and decreases blood glucose in type 2 diabetes

Supplementary Material

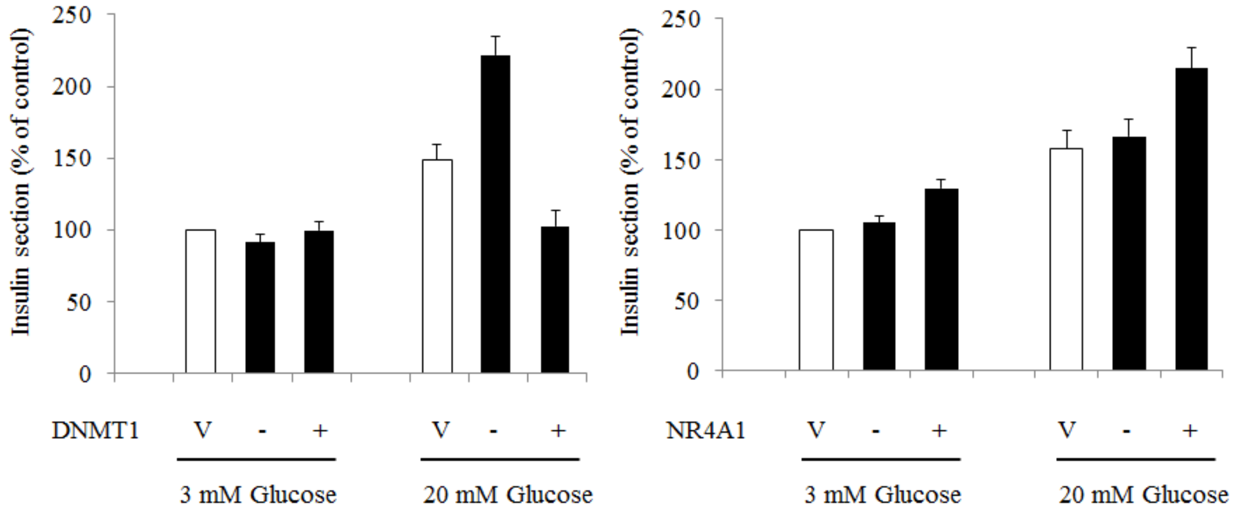

Figure S1. Effects of DNMT1 and NR4A1 expression on glucose-stimulated insulin secretion (GSIS). To examine whether DNMT1 and NR4A1 directly enhanced GSIS in cells, we cultured RIN-m5F cells in basal (3 mM) or high (20 mM) glucose in cells exhibiting overexpression or knockdown of DNMT1 and NR4A1. The results showed the high glucose significantly induced insulin secretion, whereas the GSIS was further increased in NR4A1-overexpressing cells and DNMT1-knockdown cells.

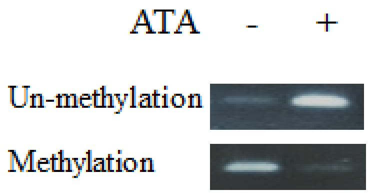

Figure S2. ATA decreased NR4A1 DNA hypermethylation in yKK mice. After ATA treatment, DNA hypermethylation was decreased in yKK mice.

MATERIALS AND METHODS

Glucose-stimulated insulin secretion (GSIS). RIN-m5F cells were culture in 6-well plates in RPMI medium containing 11.1 mM glucose and 10 % FBS at 37°C until cells became 50–60% confluent. The culture was subcultured to medium containing 3 mM glucose for 12 h. To determine the effects of NR4A1 and DNMT1 overexpression or knockdown on GSIS, cells were washed with Krebs-Ringer bicarbonate (KRB) buffer (129 mM NaCl, 1.2 mM $MgSO_4$, 1.2 mM KH_2PO_4, 2.5 mM $CaCl_2$, 5 mM $NaHCO_3$, and 0.1% BSA, pH 7.4) followed by incubation in KRB buffer containing 3 or 20 mM glucose in the context of NR4A1 and DNMT1 overexpression or knockdown for 48 h. Insulin secretion in supernatants was measured using an Insulin Human ELISA Kit (Abcam, MA,USA).

NR4A1 DNA methylation polymerase chain reaction. Genomic DNA was extracted from murine peripheral blood leukocytes using a Genomic DNA extraction kit (Qiagen, Valencia, CA, USA), according to the manufacturer's instructions. Bisulfite modification of genomic DNA was carried out using an EZ DNA methylation Kit (Zymo Research, Irvine, CA, USA). Primer sequences used for NR4A1 MSP were as follows: F: 5′-GTTATTTTTA GTTTATTGATGAGGTTG-3′, R: 5′-AAAAATTCATCCATACAAACCACC-3′.
